# Physiological and pathological roles of tissue plasminogen activator and its inhibitor neuroserpin in the nervous system

**DOI:** 10.3389/fncel.2015.00396

**Published:** 2015-10-13

**Authors:** Tet Woo Lee, Vicky W. K. Tsang, Nigel P. Birch

**Affiliations:** ^1^School of Biological Sciences and Centre for Brain Research, University of AucklandAuckland, New Zealand; ^2^Brain Research New Zealand, Rangahau Roro AotearoaAuckland, New Zealand

**Keywords:** serine protease, serpin, neuronal migration, neurite growth, synaptic plasticity, neurodegeneration and neuroprotection, Alzheimer's disease, neurovascular unit

## Abstract

Although its roles in the vascular space are most well-known, tissue plasminogen activator (tPA) is widely expressed in the developing and adult nervous system, where its activity is believed to be regulated by neuroserpin, a predominantly brain-specific member of the serpin family of protease inhibitors. In the normal physiological state, tPA has been shown to play roles in the development and plasticity of the nervous system. Ischemic damage, however, may lead to excess tPA activity in the brain and this is believed to contribute to neurodegeneration. In this article, we briefly review the physiological and pathological roles of tPA in the nervous system, which includes neuronal migration, axonal growth, synaptic plasticity, neuroprotection and neurodegeneration, as well as a contribution to neurological disease. We summarize tPA's multiple mechanisms of action and also highlight the contributions of the inhibitor neuroserpin to these processes.

## Introduction

**Figure 1:**
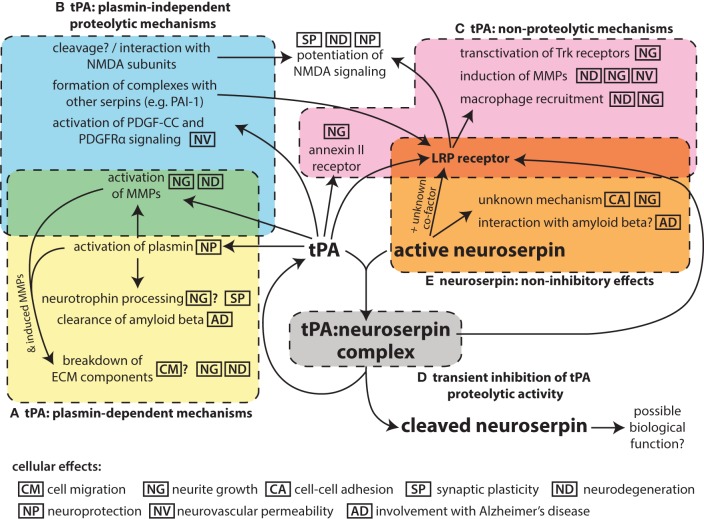
**Schematic summarizing the pleotropic roles of tPA in the nervous system and its interaction with the inhibitor neuroserpin**. While tPA is named for its function to proteolytically activate the zymogen plasminogen to plasmin **(A)**, it can also act on other substrates in a plasmin-independent manner **(B)**. In addition, tPA can bind to cell-surface receptors and act via non-proteolytic mechanisms **(C)**, although binding to the LRP receptor in particular is affected by formation of complexes of tPA with serpin partners, which requires the proteolytic activity of tPA. The proteolytic activity of tPA can be inhibited by neuroserpin **(D)**, although tPA:neuroserpin complexes are unstable and this inhibition is only transient. There is also evidence of neuroserpin having non-inhibitory effects **(E)** although the mechanism of these is unknown. The main molecular events for each mechanism are indicated; the icons indicate the cellular effects associated with these molecular events. Effects for which there is only limited evidence are shown with a question mark.

Research of tissue plasminogen activator (tPA) in the nervous system has linked this protease to a number of functions, including cell migration, axonal growth, and synaptic plasticity, as well as a contribution to neurodegeneration in pathological states. The main inhibitor of plasminogen activator proteolytic activity in the vascular space is the serpin plasminogen activator inhibitor 1 (PAI-1; SERPINE1). This serpin, however, is only weakly expressed in the brain (Sawdey and Loskutoff, 1991; Masos and Miskin, 1997). Another serpin, protease nexin-1 (PN-1; SERPINE2) is expressed throughout the brain (Sappino et al., 1993; Reinhard et al., 1994). Although PN-1 may play some role in regulating tPA activity (Kvajo et al., 2004; Samson et al., 2008), its inhibitory kinetics suggest that it mainly functions as inhibitor of thrombin (Scott et al., 1985). Instead, the predominant inhibitor of neuronal tPA activity is believed to be the neuroserpin (SERPINI1), a serpin that is largely specific to the nervous system (Osterwalder et al., 1996; Hastings et al., 1997; Krueger et al., 1997). This review will focus on the interplay of these two players in the nervous system (Figure 1).

## Neuroserpin as an inhibitor of tPA

Analysis of neuroserpin sequence indicated that it was likely to be an inhibitor of trypsin-like serine proteases (Osterwalder et al., 1996). Biochemical evidence subsequently showed strong inhibition of tPA by neuroserpin and considerably less efficient inhibition of urokinase plasminogen activator (uPA), trypsin, NGF-γ, plasmin, and thrombin (Hastings et al., 1997; Osterwalder et al., 1998). The function of neuroserpin as an inhibitor of tPA is supported by their similar expression patterns in the nervous system (Hastings et al., 1997; Krueger et al., 1997; Teesalu et al., 2004) and data showing that tPA activity levels are decreased by over-expression of neuroserpin in the brain (Cinelli et al., 2001).

Other results, however, indicate that neuroserpin does not behave as a classical inhibitory serpin toward tPA. Unlike most covalent serpin:protease complexes, tPA:neuroserpin is unstable and dissociates within minutes to release cleaved neuroserpin and active tPA (Barker-Carlson et al., 2002; Ricagno et al., 2009; Lee et al., 2015). As complex dissociation is expected to occur prior to clearance (Barker-Carlson et al., 2002), these data suggest that neuroserpin is likely to function as a transient inhibitor of tPA *in vivo*. Interestingly, evolutionarily conserved residues in neuroserpin regulate the half-life of tPA:neuroserpin complexes, suggesting that the precise half-life of the complexes may be physiologically important (Lee et al., 2015). tPA:neuroserpin interactions may involve other players, such as an unknown co-factor that stabilizes the complex (Barker-Carlson et al., 2002). Neuroserpin's weak inhibition of other proteases, such as plasmin, may also be physiological important (Wu et al., 2010) and it is also possible that neuroserpin may have other protease targets that have not yet been determined. There is evidence of non-inhibitory functions of neuroserpin (Lee et al., 2008), raising the alternate possibility that tPA modulates neuroserpin activity by cleaving neuroserpin to produce a form with distinct (non-inhibitory) biological activity.

## Expression of neuroserpin and tPA in the nervous system

Both tPA and neuroserpin are expressed in neurons throughout the developing and the adult nervous systems (Sappino et al., 1993; Friedman and Seeds, 1994; Ware et al., 1995; Osterwalder et al., 1996; Krueger et al., 1997; Teesalu et al., 2004), with spatial and temporal expression patterns suggesting roles in neuronal migration, axonal growth, synaptic development, neuronal plasticity and regulation of neurovascular responses. High neuroserpin expression has been shown in post-mitotic cells undergoing neurogenesis in the adult (Yamada et al., 2010), also suggesting a function in neuronal maturation.

Neuroserpin and tPA are targeted to the regulated secretory pathway, being sorted to dense core secretory granules and released in response to stimulation (Parmer et al., 1997; Lochner et al., 1998; Hill et al., 2000; Parmar et al., 2002; Silverman et al., 2005; Ishigami et al., 2007; Miranda et al., 2008). At a subcellular level, tPA has been localized to neuronal growth cones (Lochner et al., 1998; Silverman et al., 2005) and dendritic spines (Lochner et al., 2006), while neuroserpin has been localized to the neurite tips of differentiated PC12 cells (Parmar et al., 2002; Miranda et al., 2008), as well as axons, dendrites and presynaptic terminals of cultured neurons (Ishigami et al., 2007; Borges et al., 2010). Two unique features of neuroserpin that are important for regulated secretion are a targeting sequence at the C-terminus (Ishigami et al., 2007) and a resistance to polymerization at low pH (Belorgey et al., 2010).

In addition to activity-dependent secretion, it has been shown that the expression of tPA is regulated by several forms of neuronal activity including long-term potentiation (LTP) (Qian et al., 1993) and long term depression (LTD) (Napolitano et al., 1999; Calabresi et al., 2000). The expression of neuroserpin has also been shown to be regulated by neuronal depolarization (Berger et al., 1999), neuronal activity during visual cortex development (Wannier-Morino et al., 2003), and several signaling factors and hormones including nerve growth factor (NGF), anti-Müllerian hormone (AMH), thyroid hormone, and progesterone (Berger et al., 1999; Navarro-Yubero et al., 2004; Lebeurrier et al., 2008; Vanlandingham et al., 2008).

## Functions of tPA and neuroserpin in neuronal migration and axonal growth

A role for tPA in neuronal migration is supported by results showing that migration of cerebellar granule neurons is perturbed in tPA-deficient mice (Seeds et al., 1999). While it has been hypothesized that tPA regulates neuronal migration by activating plasmin to break down cell adhesions or extracellular matrix (ECM) (Seeds et al., 1999; Basham and Seeds, 2001), there is no direct evidence to support this role.

Evidence for a function of tPA and neuroserpin in regulating axonal growth has come from studies of cultured cells. Inhibition of tPA activity or tPA knockout have been shown to block axonal growth in cultured neurons (Pittman et al., 1989; Baranes et al., 1998; Minor et al., 2009), while exogenous tPA or tPA over-expression causes increased neurite outgrowth (Pittman and Dibenedetto, 1995; Baranes et al., 1998; Lee et al., 2007a). Similarly, altered expression of neuroserpin has been shown to trigger changes in the extension of neurite-like processes of AtT-20 cells (Hill et al., 2000) and NGF-mediated neurite outgrowth in PC12 cells (Parmar et al., 2002; Navarro-Yubero et al., 2004).

A role of tPA in axonal growth has also been shown *in vivo*. In one study, tPA-knockout mice show abnormal growth of mossy fiber axons in the dentate gyrus following seizure (Wu et al., 2000). Other reports have focused on the role of tPA in axonal regeneration following damage. In studies using the sciatic nerve crush model of peripheral nervous system regeneration, tPA is induced in the neurons and supporting cells of the nerve following crush damage, while axonal regeneration and functional recovery is reduced in tPA or plasminogen knockout animals and improved with exogenous tPA or tPA/plasminogen (Akassoglou et al., 2000; Siconolfi and Seeds, 2001, 2003; Zou et al., 2006).

Multiple mechanisms have been suggested to mediate the effects of tPA on axonal growth. Proteolysis of ECM components may create channels for neurites to extend through (Pittman and Dibenedetto, 1995) and/or remove the inhibitory effects of these components (Wu et al., 2000; Bukhari et al., 2011). This is likely to involve activation or induction of additional downstream proteases such as matrix metalloproteinases (MMPs) (Siconolfi and Seeds, 2003; Wang et al., 2003; Hu et al., 2006; Zou et al., 2006). During axonal regeneration, the removal of fibrin deposits by tPA/plasmin also appears to be important (Akassoglou et al., 2000; Zou et al., 2006), as well as macrophage recruitment to remove cellular debris (Zou et al., 2006), which may involve tPA binding to the LDL-related protein (LRP) receptor (Cao et al., 2006). Binding of tPA to the LRP receptor and the annexin II receptor has also been shown to mediate non-proteolytic effects of tPA on neurite outgrowth (Lee et al., 2007a; Shi et al., 2009). Finally, tPA may regulate neurite growth via proteolytic processing of neurotrophins (Pang et al., 2004; Bruno and Cuello, 2006).

The mechanism of neuroserpin's effects on neurite outgrowth are largely undetermined. Neuroserpin may act by modulating tPA activity, for example, neuroserpin has been shown to regulate proteolytic processing of the neurotrophin NGF (Bruno and Cuello, 2006). Interestingly, the neurite outgrowth effects of neuroserpin could be triggered by non-inhibitory mutant forms of neuroserpin (Lee et al., 2008), suggesting neuroserpin may also act independently of tPA, possibly by binding to a cell surface receptor such as LRP (Makarova et al., 2003).

## Effects of tPA and neuroserpin on neuronal plasticity

Deficits in hippocampal late phase LTP are seen in tPA-knockout mice (Frey et al., 1996; Huang et al., 1996; Calabresi et al., 2000). Conversely, LTP is increased by exogenous tPA or tPA overexpression (Baranes et al., 1998; Madani et al., 1999). Knockout of the tPA gene also leads to defects in both LTP and LTD in the striatum (Calabresi et al., 2000; Centonze et al., 2002). Numerous studies have also shown a role of tPA in memory and learning. For example, tPA-knockout mice exhibit deficits in hippocampal-dependent and striatum-dependent tasks (Huang et al., 1996; Calabresi et al., 2000; Pawlak et al., 2002; Benchenane et al., 2007) and cerebellar motor learning (Seeds et al., 2003), while transgenic mice over-expressing tPA were found to have improved spatial learning (Madani et al., 1999). tPA is also required for altered amygdala- and hippocampal-dependent behavioral responses that occur in mice subjected to restraint-stress (Pawlak et al., 2003, 2005b; Norris and Strickland, 2007).

At the cellular level, tPA's involvement in LTP has been associated with the formation of new presynaptic varicosities (Baranes et al., 1998), while activity-dependent formation of perforated synapses in cultured neurons can be blocked by tPA inhibitors (Neuhoff et al., 1999). In animals subjected to restraint stress, induction of the plasticity-related gene GAP43 (Pawlak et al., 2003) and changes in dendritic spine number were absent in tPA knockout mice (Pawlak et al., 2005b). During visual cortex development, experience-dependent plasticity and pruning of dendritic spines is also reduced in tPA-knockout mice and can be partly restored by exogenous tPA (Mataga et al., 2002, 2004).

A number of different mechanism underlying tPA's effects on synaptic plasticity have been proposed. Firstly, tPA may contribute to LTP by regulating plasmin-mediated processing of BDNF from its precursor proBDNF to mature BDNF (mBDNF) (Pang et al., 2004; Barnes and Thomas, 2008). It has been shown that tPA is secreted from neurons in response to high-frequency, but not low-frequency, stimulation of neurons, leading to changes in the proBDNF/mBDNF ratio (Nagappan et al., 2009). Since proBDNF has been linked to LTD (Woo et al., 2005) while mBDNF has been linked to LTP, these results suggest that tPA may mediate the differing cellular responses to different patterns of neuronal activity. Secondly, there is general agreement in the literature that tPA can potentiate NMDA-receptor signaling. The manner in which it does so, however, remains unclear. Results from the Vivien group suggest that tPA may act by interacting with the GluN1 subunit of NMDA receptors, particularly in GluN2D-containing receptors (Benchenane et al., 2007; Macrez et al., 2010; Obiang et al., 2012). Other results suggest that tPA modulates NMDA signaling through GluN2B subunits (Pawlak et al., 2005a; Norris and Strickland, 2007; Noel et al., 2011; Ng et al., 2012) or by a mechanism involving LRP (Martin et al., 2008; Samson et al., 2008). The importance of LRP in tPA-mediated neuronal plasticity was also reported in an earlier study on LTP (Zhuo et al., 2000).

Experiments in culture systems have provided some evidence that neuroserpin is involved in cellular plasticity. Firstly, altered neuroserpin expression in PC12 cells has been linked to changes in cell-cell adhesion mediated by the synaptic adhesion molecule N-cadherin (Lee et al., 2008). In addition, overexpression of neuroserpin in cultured neurons has been found to lead to changes in the number and shape of dendritic spines (Borges et al., 2010). Altered neuroserpin expression *in vivo* has also been shown to lead to behavioral changes, with both neuroserpin overexpression and neuroserpin-knockout leading to increased phobic and anxiety-like responses (Madani et al., 2003). Localized overexpression of neuroserpin in the adult rat hippocampus did not cause any changes in learning and memory, but it altered the expression of postsynaptic scaffolding protein PSD-95 (Tsang et al., 2014). Overall, little is known about neuroserpin's mechanism of action for these effects, however, the results from the PC12 studies show that inhibition of tPA was not required (Lee et al., 2008), and the changes in behavior in neuroserpin-knockout animals were not correlated with altered tPA activity (Madani et al., 2003).

## Neuroserpin and tPA in neurodegeneration and neuroprotection

Initial evidence for a contribution of tPA to neuronal death came some years ago, when it was shown that tPA knockout mice were resistant to excitotoxin-induced neuronal degeneration (Tsirka et al., 1995) and had reduced ischemic damage in a stroke model (Wang et al., 1998). These results have been independently confirmed by a number of other groups (Strickland, 2001; Kaur et al., 2004).

Three main mechanisms for tPA's effects on neuronal death have been proposed. Firstly, tPA may cause ECM breakdown by proteolytically activating plasmin and/or MMPs (Chen and Strickland, 1997; Tsirka et al., 1997; Sumii and Lo, 2002; Wang et al., 2003). Secondly, the ability of tPA to potentiate NMDA receptor-mediated calcium influx may also contribute by promoting excitotoxic neuronal death (Nicole et al., 2001). In support of this, immunotherapy to block interaction of tPA with NMDA receptors has been shown to reduce neuronal damage in stroke models (Benchenane et al., 2007; Gaberel et al., 2013). Thirdly, tPA may signal through the LRP receptor to trigger a number of inter-related effects including induction of MMP expression (Wang et al., 2003, 2004; Lee et al., 2007b; Sashindranath et al., 2012), opening of the blood-brain barrier (Yepes et al., 2003; Sashindranath et al., 2012) and recruitment and activation of microglia (Rogove and Tsirka, 1998; Rogove et al., 1999; Siao and Tsirka, 2002; Zhang et al., 2009). Paradoxically, the tPA inhibitor PAI-1 has been shown to exacerbate, rather than reduce, some of these effects of tPA, as tPA:PAI-1 complexes bind more strongly to LRP than tPA itself (Sashindranath et al., 2012). The instability of tPA:neuroserpin complexes could therefore be a mechanism to temporarily reduce tPA activity without excessive LRP activation.

There is also evidence of neuroprotective effects of tPA, first shown some time ago (Kim et al., 1999; Yi et al., 2004; Liot et al., 2006) but highlighted by a series of recent results from the *in vitro* oxygen and glucose deprivation (OGD) model of ischemic death, as well as in *in vivo* models of excitotoxic neuronal death (Haile et al., 2012; Wu et al., 2012, 2013). These studies have suggested that lower concentrations of tPA mediate survival instead of neuronal death, through both plasmin-dependent and LRP-dependent/plasmin-independent mechanisms involving NMDA signaling.

In animal models of stroke, administration of exogenous neuroserpin alone, neuroserpin in combination with tPA and neuroserpin overexpression have been shown to reduce ischemic damage *in vivo* (Yepes et al., 2000; Cinelli et al., 2001; Zhang et al., 2002). In these studies, the effects of neuroserpin were associated with reductions in tPA and uPA activity, ECM degradation, microglia activation and blood brain barrier leakage. Conversely, neuroserpin-knockout mice have worse ischemic damage and neurological outcomes than controls, with the effects attributed to tPA-mediated activation of microglia (Gelderblom et al., 2013). Similarly, studies in the OGD model and a mouse model of motoneuropathy have also shown neuroprotective effects of neuroserpin with results suggesting a mechanism involving tPA inhibition (Simonin et al., 2006; Rodríguez-González et al., 2011). However, neuroserpin has been shown to promote neuronal survival in tPA knockout mice, indicating it can also act through a tPA-independent mechanism, possibly through inhibition of uPA or plasmin (Wu et al., 2010).

## Functions of tPA and neuroserpin in the neurovascular unit

There is considerable evidence that tPA in the central nervous system side of the neurovascular unit increases the permeability of the blood-brain barrier (e.g., Yepes et al., 2003; Su et al., 2008; Sashindranath et al., 2012). This effect of tPA may contribute to neurodegeneration following stroke, and recent results also suggest a contribution to seizure propagation (Fredriksson et al., 2015). tPA has also been shown to regulate functional hyperemia (Park et al., 2008). A number of downstream events have been identified for the neurovascular effects of tPA including activation of neuronal nitric oxide synthase (Parathath et al., 2006; Park et al., 2008), proteolytic activation of platelet-derived growth factor-CC (PDGF-CC) and platelet-derived growth factor receptor alpha (PDGFRα) signaling (Su et al., 2008; Fredriksson et al., 2015), LRP signaling and induction of MMPs (Sashindranath et al., 2012). While most of the neurovascular effects of tPA are considered to be plasmin-independent (Yepes et al., 2003), there is also evidence for an involvement of plasmin (Freeman et al., 2014; Niego and Medcalf, 2014). As an inhibitor of tPA, neuroserpin can act as an antagonist of tPA in the neurovascular unit (Yepes et al., 2003; Fredriksson et al., 2015).

## Contributions of tPA and neuroserpin to neurological disease

Mutations in the neuroserpin gene cause a rare autosomal-dominant dementia accompanied by epilepsy called Familial Encephalopathy with Neuroserpin Inclusion Bodies (FENIB), characterized by polymerization of neuroserpin, formation of inclusion bodies and subsequent neuronal degeneration (Davis et al., 1999, 2002; Takao et al., 2000; Gourfinkel-An et al., 2007; Coutelier et al., 2008; Hagen et al., 2011). Other studies have suggested a role for neuroserpin in Alzheimer's disease, with neuroserpin hypothesized to be either beneficial by interacting with amyloid-beta peptides and altering their oligomerization (Kinghorn et al., 2006) or detrimental by reducing tPA-mediated clearance of amyloid-beta (Fabbro and Seeds, 2009; Fabbro et al., 2011). Changes in the expression of neuroserpin have also been linked to schizophrenia (Hakak et al., 2001; Vawter et al., 2004; Brennand et al., 2011). A recent study also suggests that the expression of neuroserpin by tumor cells may inhibit plasmin-mediated death signals and allow metastasis into the brain (Valiente et al., 2014).

## Conclusion

Research over the years has shown that tPA has pleiotropic effects in the nervous system and can act through multiple mechanisms. It is also clear that neuroserpin does not function as a classical serpin inhibitor for tPA and this must be considered when making inferences regarding its function and mode of action. Future research should take a broad view and consider all possible mechanisms of these two players to provide a more complete understanding of their roles in the nervous system.

### Conflict of interest statement

The authors declare that the research was conducted in the absence of any commercial or financial relationships that could be construed as a potential conflict of interest.

## References

[B1] Akassoglou K. Kombrinck K. W. Degen J. L. Strickland S. (2000). Tissue plasminogen activator-mediated fibrinolysis protects against axonal degeneration and demyelination after sciatic nerve injury. J. Cell Biol. 149, 1157–1166. 10.1083/jcb.149.5.1157 10831618 PMC2174825

[B2] Baranes D. Lederfein D. Huang Y. Y. Chen M. Bailey C. H. Kandel E. R. (1998). Tissue plasminogen activator contributes to the late phase of LTP and to synaptic growth in the hippocampal mossy fiber pathway. Neuron 21, 813–825. 10.1016/S0896-6273(00)80597-8 9808467

[B3] Barker-Carlson K. Lawrence D. A. Schwartz B. S. (2002). Acyl-enzyme complexes between tissue-type plasminogen activator and neuroserpin are short-lived *in vitro*. J. Biol. Chem. 277, 46852–46857. 10.1074/jbc.M207740200 12228252

[B4] Barnes P. Thomas K. L. (2008). Proteolysis of proBDNF is a key regulator in the formation of memory. PLoS ONE 3:e3248. 10.1371/journal.pone.0003248 18813339 PMC2532744

[B5] Basham M. E. Seeds N. W. (2001). Plasminogen expression in the neonatal and adult mouse brain. J. Neurochem. 77, 318–325. 10.1046/j.1471-4159.2001.t01-1-00239.x 11279287

[B6] Belorgey D. Hägglöf P. Onda M. Lomas D. A. (2010). pH-dependent stability of neuroserpin is mediated by histidines 119 and 138; implications for the control of beta-sheet A and polymerization. Protein Sci. 19, 220–228. 10.1002/pro.299 19953505 PMC2865726

[B7] Benchenane K. Castel H. Boulouard M. Bluthé R. Fernandez-Monreal M. Roussel B. D.. (2007). Anti-NR1 N-terminal-domain vaccination unmasks the crucial action of tPA on NMDA-receptor-mediated toxicity and spatial memory. J. Cell Sci. 120, 578–585. 10.1242/jcs.03354 17244650

[B8] Berger P. Kozlov S. V. Cinelli P. Krüger S. R. Vogt L. Sonderegger P. (1999). Neuronal depolarization enhances the transcription of the neuronal serine protease inhibitor neuroserpin. Mol. Cell. Neurosci. 14, 455–467. 10.1006/mcne.1999.0804 10656253

[B9] Borges V. M. Lee T. W. Christie D. L. Birch N. P. (2010). Neuroserpin regulates the density of dendritic protrusions and dendritic spine shape in cultured hippocampal neurons. J. Neurosci. Res. 88, 2610–2617. 10.1002/jnr.22428 20648651

[B10] Brennand K. J. Simone A. Jou J. Gelboin-Burkhart C. Tran N. Sangar S.. (2011). Modelling schizophrenia using human induced pluripotent stem cells. Nature 473, 221–225. 10.1038/nature09915 21490598 PMC3392969

[B11] Bruno M. A. Cuello A. C. (2006). Activity-dependent release of precursor nerve growth factor, conversion to mature nerve growth factor, and its degradation by a protease cascade. Proc. Natl. Acad. Sci. U.S.A. 103, 6735–6740. 10.1073/pnas.0510645103 16618925 PMC1458950

[B12] Bukhari N. Torres L. Robinson J. K. Tsirka S. E. (2011). Axonal regrowth after spinal cord injury via chondroitinase and the tissue plasminogen activator (tPA)/plasmin system. J. Neurosci. 31, 14931–14943. 10.1523/JNEUROSCI.3339-11.2011 22016526 PMC3206287

[B13] Calabresi P. Napolitano M. Centonze D. Marfia G. A. Gubellini P. Teule M. A.. (2000). Tissue plasminogen activator controls multiple forms of synaptic plasticity and memory. Eur. J. Neurosci. 12, 1002–1012. 10.1046/j.1460-9568.2000.00991.x 10762331

[B14] Cao C. Lawrence D. A. Li Y. Von Arnim C. A. Herz J. Su E. J.. (2006). Endocytic receptor LRP together with tPA and PAI-1 coordinates Mac-1-dependent macrophage migration. EMBO J. 25, 1860–1870. 10.1038/sj.emboj.7601082 16601674 PMC1456942

[B15] Centonze D. Napolitano M. Saulle E. Gubellini P. Picconi B. Martorana A.. (2002). Tissue plasminogen activator is required for corticostriatal long-term potentiation. Eur. J. Neurosci. 16, 713–721. 10.1046/j.1460-9568.2002.02106.x 12270047

[B16] Chen Z. L. Strickland S. (1997). Neuronal death in the hippocampus is promoted by plasmin-catalyzed degradation of laminin. Cell 91, 917–925. 10.1016/S0092-8674(00)80483-3 9428515

[B17] Cinelli P. Madani R. Tsuzuki N. Vallet P. Arras M. Zhao C. N.. (2001). Neuroserpin, a neuroprotective factor in focal ischemic stroke. Mol. Cell. Neurosci. 18, 443–457. 10.1006/mcne.2001.1028 11922137

[B18] Coutelier M. Andries S. Ghariani S. Dan B. Duyckaerts C. Van Rijckevorsel K.. (2008). Neuroserpin mutation causes electrical status epilepticus of slow-wave sleep. Neurology 71, 64–66. 10.1212/01.wnl.0000316306.08751.28 18591508

[B19] Davis R. L. Shrimpton A. E. Carrell R. W. Lomas D. A. Gerhard L. Baumann B.. (2002). Association between conformational mutations in neuroserpin and onset and severity of dementia. Lancet 359, 2242–2247. 10.1016/S0140-6736(02)09293-0 12103288

[B20] Davis R. L. Shrimpton A. E. Holohan P. D. Bradshaw C. Feiglin D. Collins G. H.. (1999). Familial dementia caused by polymerization of mutant neuroserpin. Nature 401, 376–379. 10.1038/43894 10517635

[B21] Fabbro S. Schaller K. Seeds N. W. (2011). Amyloid-beta levels are significantly reduced and spatial memory defects are rescued in a novel neuroserpin-deficient Alzheimer's disease transgenic mouse model. J. Neurochem. 118, 928–938. 10.1111/j.1471-4159.2011.07359.x 21689108

[B22] Fabbro S. Seeds N. W. (2009). Plasminogen activator activity is inhibited while neuroserpin is up-regulated in the Alzheimer disease brain. J. Neurochem. 109, 303–315. 10.1111/j.1471-4159.2009.05894.x 19222708

[B23] Fredriksson L. Stevenson T. K. Su E. J. Ragsdale M. Moore S. Craciun S.. (2015). Identification of a neurovascular signaling pathway regulating seizures in mice. Ann. Clin. Transl. Neurol. 2, 722–738. 10.1002/acn3.209 26273685 PMC4531055

[B24] Freeman R. Niego B. Croucher D. R. Pedersen L. O. Medcalf R. L. (2014). t-PA, but not desmoteplase, induces plasmin-dependent opening of a blood-brain barrier model under normoxic and ischaemic conditions. Brain Res. 1565, 63–73. 10.1016/j.brainres.2014.03.027 24675027

[B25] Frey U. Müller M. Kuhl D. (1996). A different form of long-lasting potentiation revealed in tissue plasminogen activator mutant mice. J. Neurosci. 16, 2057–2063. 8604050 10.1523/JNEUROSCI.16-06-02057.1996 PMC6578503

[B26] Friedman G. C. Seeds N. W. (1994). Tissue plasminogen activator expression in the embryonic nervous system. Brain Res. Dev. Brain Res. 81, 41–49. 10.1016/0165-3806(94)90066-3 7805285

[B27] Gaberel T. Macrez R. Gauberti M. Montagne A. Hebert M. Petersen K. U.. (2013). Immunotherapy blocking the tissue plasminogen activator-dependent activation of N-methyl-D-aspartate glutamate receptors improves hemorrhagic stroke outcome. Neuropharmacology 67, 267–271. 10.1016/j.neuropharm.2012.11.023 23219658

[B28] Gelderblom M. Neumann M. Ludewig P. Bernreuther C. Krasemann S. Arunachalam P.. (2013). Deficiency in serine protease inhibitor neuroserpin exacerbates ischemic brain injury by increased postischemic inflammation. PLoS ONE 8:e63118. 10.1371/journal.pone.0063118 23658802 PMC3643909

[B29] Gourfinkel-An I. Duyckaerts C. Camuzat A. Meyrignac C. Sonderegger P. Baulac M.. (2007). Clinical and neuropathologic study of a French family with a mutation in the neuroserpin gene. Neurology 69, 79–83. 10.1212/01.wnl.0000265052.99144.b5 17606885

[B30] Hagen M. C. Murrell J. R. Delisle M. B. Andermann E. Andermann F. Guiot M. C.. (2011). Encephalopathy with neuroserpin inclusion bodies presenting as progressive myoclonus epilepsy and associated with a novel mutation in the Proteinase Inhibitor 12 gene. Brain Pathol. 21, 575–582. 10.1111/j.1750-3639.2011.00481.x 21435071 PMC3709456

[B31] Haile W. B. Wu J. Echeverry R. Wu F. An J. Yepes M. (2012). Tissue-type plasminogen activator has a neuroprotective effect in the ischemic brain mediated by neuronal TNF-alpha. J. Cereb. Blood Flow Metab. 32, 57–69. 10.1038/jcbfm.2011.106 21792242 PMC3323291

[B32] Hakak Y. Walker J. R. Li C. Wong W. H. Davis K. L. Buxbaum J. D.. (2001). Genome-wide expression analysis reveals dysregulation of myelination-related genes in chronic schizophrenia. Proc. Natl. Acad. Sci. U.S.A. 98, 4746–4751. 10.1073/pnas.081071198 11296301 PMC31905

[B33] Hastings G. A. Coleman T. A. Haudenschild C. C. Stefansson S. Smith E. P. Barthlow R.. (1997). Neuroserpin, a brain-associated inhibitor of tissue plasminogen activator is localized primarily in neurons. Implications for the regulation of motor learning and neuronal survival. J. Biol. Chem. 272, 33062–33067. 10.1074/jbc.272.52.33062 9407089

[B34] Hill R. M. Parmar P. K. Coates L. C. Mezey E. Pearson J. F. Birch N. P. (2000). Neuroserpin is expressed in the pituitary and adrenal glands and induces the extension of neurite-like processes in AtT-20 cells. Biochem. J. 345(Pt 3), 595–601. 10.1042/bj3450595 10642518 PMC1220794

[B35] Hu K. Yang J. Tanaka S. Gonias S. L. Mars W. M. Liu Y. (2006). Tissue-type plasminogen activator acts as a cytokine that triggers intracellular signal transduction and induces matrix metalloproteinase-9 gene expression. J. Biol. Chem. 281, 2120–2127. 10.1074/jbc.M504988200 16303771

[B36] Huang Y. Y. Bach M. E. Lipp H. P. Zhuo M. Wolfer D. P. Hawkins R. D.. (1996). Mice lacking the gene encoding tissue-type plasminogen activator show a selective interference with late-phase long-term potentiation in both Schaffer collateral and mossy fiber pathways. Proc. Natl. Acad. Sci. U.S.A. 93, 8699–8704. 10.1073/pnas.93.16.8699 8710934 PMC38736

[B37] Ishigami S. Sandkvist M. Tsui F. Moore E. Coleman T. A. Lawrence D. A. (2007). Identification of a novel targeting sequence for regulated secretion in the serine protease inhibitor neuroserpin. Biochem. J. 402, 25–34. 10.1042/BJ20061170 17040209 PMC1783992

[B38] Kaur J. Zhao Z. Klein G. M. Lo E. H. Buchan A. M. (2004). The neurotoxicity of tissue plasminogen activator? J. Cereb. Blood Flow Metab. 24, 945–963. 10.1097/01.WCB.0000137868.50767.E8 15356416

[B39] Kim Y. H. Park J. H. Hong S. H. Koh J. Y. (1999). Nonproteolytic neuroprotection by human recombinant tissue plasminogen activator. Science 284, 647–650. 10.1126/science.284.5414.647 10213688

[B40] Kinghorn K. J. Crowther D. C. Sharp L. K. Nerelius C. Davis R. L. Chang H. T.. (2006). Neuroserpin binds Abeta and is a neuroprotective component of amyloid plaques in Alzheimer disease. J. Biol. Chem. 281, 29268–29277. 10.1074/jbc.M600690200 16849336

[B41] Krueger S. R. Ghisu G. P. Cinelli P. Gschwend T. P. Osterwalder T. Wolfer D. P.. (1997). Expression of neuroserpin, an inhibitor of tissue plasminogen activator, in the developing and adult nervous system of the mouse. J. Neurosci. 17, 8984–8996. 9364046 10.1523/JNEUROSCI.17-23-08984.1997 PMC6573583

[B42] Kvajo M. Albrecht H. Meins M. Hengst U. Troncoso E. Lefort S.. (2004). Regulation of brain proteolytic activity is necessary for the *in vivo* function of NMDA receptors. J. Neurosci. 24, 9734–9743. 10.1523/JNEUROSCI.3306-04.2004 15509762 PMC6730139

[B43] Lebeurrier N. Launay S. Macrez R. Maubert E. Legros H. Leclerc A.. (2008). Anti-Mullerian-hormone-dependent regulation of the brain serine-protease inhibitor neuroserpin. J. Cell Sci. 121, 3357–3365. 10.1242/jcs.031872 18796535

[B44] Lee H. Y. Hwang I. Y. Im H. Koh J. Y. Kim Y. H. (2007a). Non-proteolytic neurotrophic effects of tissue plasminogen activator on cultured mouse cerebrocortical neurons. J. Neurochem. 101, 1236–1247. 10.1111/j.1471-4159.2007.04417.x 17498240

[B45] Lee S. R. Lok J. Rosell A. Kim H. Y. Murata Y. Atochin D.. (2007b). Reduction of hippocampal cell death and proteolytic responses in tissue plasminogen activator knockout mice after transient global cerebral ischemia. Neuroscience 150, 50–57. 10.1016/j.neuroscience.2007.06.029 17936515 PMC3814181

[B46] Lee T. W. Coates L. C. Birch N. P. (2008). Neuroserpin regulates N-cadherin-mediated cell adhesion independently of its activity as an inhibitor of tissue plasminogen activator. J. Neurosci. Res. 86, 1243–1253. 10.1002/jnr.21592 18092357

[B47] Lee T. W. Yang A. S. Brittain T. Birch N. P. (2015). An analysis approach to identify specific functional sites in orthologous proteins using sequence and structural information: application to neuroserpin reveals regions that differentially regulate inhibitory activity. Proteins 83, 135–152. 10.1002/prot.24711 25363759

[B48] Liot G. Roussel B. D. Lebeurrier N. Benchenane K. López-Atalaya J. P. Vivien D.. (2006). Tissue-type plasminogen activator rescues neurones from serum deprivation-induced apoptosis through a mechanism independent of its proteolytic activity. J. Neurochem. 98, 1458–1464. 10.1111/j.1471-4159.2006.03982.x 16800849

[B49] Lochner J. E. Honigman L. S. Grant W. F. Gessford S. K. Hansen A. B. Silverman M. A.. (2006). Activity-dependent release of tissue plasminogen activator from the dendritic spines of hippocampal neurons revealed by live-cell imaging. J. Neurobiol. 66, 564–577. 10.1002/neu.20250 16555239

[B50] Lochner J. E. Kingma M. Kuhn S. Meliza C. D. Cutler B. Scalettar B. A. (1998). Real-time imaging of the axonal transport of granules containing a tissue plasminogen activator/green fluorescent protein hybrid. Mol. Biol. Cell 9, 2463–2476. 10.1091/mbc.9.9.2463 9725906 PMC25514

[B51] Macrez R. Bezin L. Le Mauff B. Ali C. Vivien D. (2010). Functional occurrence of the interaction of tissue plasminogen activator with the NR1 Subunit of N-methyl-D-aspartate receptors during stroke. Stroke 41, 2950–2955. 10.1161/STROKEAHA.110.592360 20966414

[B52] Madani R. Hulo S. Toni N. Madani H. Steimer T. Muller D.. (1999). Enhanced hippocampal long-term potentiation and learning by increased neuronal expression of tissue-type plasminogen activator in transgenic mice. EMBO J. 18, 3007–3012. 10.1093/emboj/18.11.3007 10357813 PMC1171382

[B53] Madani R. Kozlov S. Akhmedov A. Cinelli P. Kinter J. Lipp H. P.. (2003). Impaired explorative behavior and neophobia in genetically modified mice lacking or overexpressing the extracellular serine protease inhibitor neuroserpin. Mol. Cell. Neurosci. 23, 473–494. 10.1016/S1044-7431(03)00077-0 12837630

[B54] Makarova A. Mikhailenko I. Bugge T. H. List K. Lawrence D. A. Strickland D. K. (2003). The low density lipoprotein receptor-related protein modulates protease activity in the brain by mediating the cellular internalization of both neuroserpin and neuroserpin-tissue-type plasminogen activator complexes. J. Biol. Chem. 278, 50250–50258. 10.1074/jbc.M309150200 14522960

[B55] Martin A. M. Kuhlmann C. Trossbach S. Jaeger S. Waldron E. Roebroek A.. (2008). The functional role of the second NPXY motif of the LRP1 beta-chain in tissue-type plasminogen activator-mediated activation of N-methyl-D-aspartate receptors. J. Biol. Chem. 283, 12004–12013. 10.1074/jbc.M707607200 18321860

[B56] Masos T. Miskin R. (1997). mRNAs encoding urokinase-type plasminogen activator and plasminogen activator inhibitor-1 are elevated in the mouse brain following kainate-mediated excitation. Brain Res. Mol. Brain Res. 47, 157–169. 10.1016/S0169-328X(97)00040-5 9221913

[B57] Mataga N. Mizuguchi Y. Hensch T. K. (2004). Experience-dependent pruning of dendritic spines in visual cortex by tissue plasminogen activator. Neuron 44, 1031–1041. 10.1016/j.neuron.2004.11.028 15603745

[B58] Mataga N. Nagai N. Hensch T. K. (2002). Permissive proteolytic activity for visual cortical plasticity. Proc. Natl. Acad. Sci. U.S.A. 99, 7717–7721. 10.1073/pnas.102088899 12032349 PMC124331

[B59] Minor K. Phillips J. Seeds N. W. (2009). Tissue plasminogen activator promotes axonal outgrowth on CNS myelin after conditioned injury. J. Neurochem. 109, 706–715. 10.1111/j.1471-4159.2009.05977.x 19220707

[B60] Miranda E. Macleod I. Davies M. J. Pérez J. Romisch K. Crowther D. C.. (2008). The intracellular accumulation of polymeric neuroserpin explains the severity of the dementia FENIB. Hum. Mol. Genet. 17, 1527–1539. 10.1093/hmg/ddn041 18267959 PMC2387220

[B61] Nagappan G. Zaitsev E. Senatorov V. V. Jr. Yang J. Hempstead B. L. Lu B. (2009). Control of extracellular cleavage of ProBDNF by high frequency neuronal activity. Proc. Natl. Acad. Sci. U.S.A. 106, 1267–1272. 10.1073/pnas.0807322106 19147841 PMC2633536

[B62] Napolitano M. Marfia G. A. Vacca A. Centonze D. Bellavia D. Di Marcotullio L.. (1999). Modulation of gene expression following long-term synaptic depression in the striatum. Brain Res. Mol. Brain Res. 72, 89–96. 10.1016/S0169-328X(99)00213-2 10521602

[B63] Navarro-Yubero C. Cuadrado A. Sonderegger P. Muñoz A. (2004). Neuroserpin is post-transcriptionally regulated by thyroid hormone. Brain Res. Mol. Brain Res. 123, 56–65. 10.1016/j.molbrainres.2003.12.018 15046866

[B64] Neuhoff H. Roeper J. Schweizer M. (1999). Activity-dependent formation of perforated synapses in cultured hippocampal neurons. Eur. J. Neurosci. 11, 4241–4250. 10.1046/j.1460-9568.1999.00856.x 10594650

[B65] Ng K. S. Leung H. W. Wong P. T. Low C. M. (2012). Cleavage of the NR2B subunit amino terminus of N-methyl-D-aspartate (NMDA) receptor by tissue plasminogen activator: identification of the cleavage site and characterization of ifenprodil and glycine affinities on truncated NMDA receptor. J. Biol. Chem. 287, 25520–25529. 10.1074/jbc.M112.374397 22610100 PMC3408171

[B66] Nicole O. Docagne F. Ali C. Margaill I. Carmeliet P. Mackenzie E. T.. (2001). The proteolytic activity of tissue-plasminogen activator enhances NMDA receptor-mediated signaling. Nat. Med. 7, 59–64. 10.1038/83358 11135617

[B67] Niego B. Medcalf R. L. (2014). Plasmin-dependent modulation of the blood-brain barrier: a major consideration during tPA-induced thrombolysis? J. Cereb. Blood Flow Metab. 34, 1283–1296. 10.1038/jcbfm.2014.99 24896566 PMC4126105

[B68] Noel M. Norris E. H. Strickland S. (2011). Tissue plasminogen activator is required for the development of fetal alcohol syndrome in mice. Proc. Natl. Acad. Sci. U.S.A. 108, 5069–5074. 10.1073/pnas.1017608108 21383198 PMC3064320

[B69] Norris E. H. Strickland S. (2007). Modulation of NR2B-regulated contextual fear in the hippocampus by the tissue plasminogen activator system. Proc. Natl. Acad. Sci. U.S.A. 104, 13473–13478. 10.1073/pnas.0705848104 17673549 PMC1948906

[B70] Obiang P. Macrez R. Jullienne A. Bertrand T. Lesept F. Ali C.. (2012). GluN2D subunit-containing NMDA receptors control tissue plasminogen activator-mediated spatial memory. J. Neurosci. 32, 12726–12734. 10.1523/JNEUROSCI.6202-11.2012 22972996 PMC6703808

[B71] Osterwalder T. Cinelli P. Baici A. Pennella A. Krueger S. R. Schrimpf S. P.. (1998). The axonally secreted serine proteinase inhibitor, neuroserpin, inhibits plasminogen activators and plasmin but not thrombin. J. Biol. Chem. 273, 2312–2321. 10.1074/jbc.273.4.2312 9442076

[B72] Osterwalder T. Contartese J. Stoeckli E. T. Kuhn T. B. Sonderegger P. (1996). Neuroserpin, an axonally secreted serine protease inhibitor. EMBO J. 15, 2944–2953. 8670795 PMC450235

[B73] Pang P. T. Teng H. K. Zaitsev E. Woo N. T. Sakata K. Zhen S.. (2004). Cleavage of proBDNF by tPA/plasmin is essential for long-term hippocampal plasticity. Science 306, 487–491. 10.1126/science.1100135 15486301

[B74] Parathath S. R. Parathath S. Tsirka S. E. (2006). Nitric oxide mediates neurodegeneration and breakdown of the blood-brain barrier in tPA-dependent excitotoxic injury in mice. J. Cell Sci. 119, 339–349. 10.1242/jcs.02734 16410551

[B75] Park L. Gallo E. F. Anrather J. Wang G. Norris E. H. Paul J.. (2008). Key role of tissue plasminogen activator in neurovascular coupling. Proc. Natl. Acad. Sci. U.S.A. 105, 1073–1078. 10.1073/pnas.0708823105 18195371 PMC2242693

[B76] Parmar P. K. Coates L. C. Pearson J. F. Hill R. M. Birch N. P. (2002). Neuroserpin regulates neurite outgrowth in nerve growth factor-treated PC12 cells. J. Neurochem. 82, 1406–1415. 10.1046/j.1471-4159.2002.01100.x 12354288

[B77] Parmer R. J. Mahata M. Mahata S. Sebald M. T. O'connor D. T. Miles L. A. (1997). Tissue plasminogen activator (t-PA) is targeted to the regulated secretory pathway. Catecholamine storage vesicles as a reservoir for the rapid release of t-PA. J. Biol. Chem. 272, 1976–1982. 10.1074/jbc.272.3.1976 8999889

[B78] Pawlak R. Magarinos A. M. Melchor J. McEwen B. Strickland S. (2003). Tissue plasminogen activator in the amygdala is critical for stress-induced anxiety-like behavior. Nat. Neurosci. 6, 168–174. 10.1038/nn998 12524546

[B79] Pawlak R. Melchor J. P. Matys T. Skrzypiec A. E. Strickland S. (2005a). Ethanol-withdrawal seizures are controlled by tissue plasminogen activator via modulation of NR2B-containing NMDA receptors. Proc. Natl. Acad. Sci. U.S.A. 102, 443–448. 10.1073/pnas.0406454102 15630096 PMC544297

[B80] Pawlak R. Nagai N. Urano T. Napiorkowska-Pawlak D. Ihara H. Takada Y.. (2002). Rapid, specific and active site-catalyzed effect of tissue-plasminogen activator on hippocampus-dependent learning in mice. Neuroscience 113, 995–1001. 10.1016/S0306-4522(02)00166-5 12182903

[B81] Pawlak R. Rao B. S. Melchor J. P. Chattarji S. McEwen B. Strickland S. (2005b). Tissue plasminogen activator and plasminogen mediate stress-induced decline of neuronal and cognitive functions in the mouse hippocampus. Proc. Natl. Acad. Sci. USA. 102, 18201–18206. 10.1073/pnas.0509232102 16330749 PMC1312427

[B82] Pittman R. N. Dibenedetto A. J. (1995). PC12 cells overexpressing tissue plasminogen activator regenerate neurites to a greater extent and migrate faster than control cells in complex extracellular matrix. J. Neurochem. 64, 566–575. 10.1046/j.1471-4159.1995.64020566.x 7830049

[B83] Pittman R. N. Ivins J. K. Buettner H. M. (1989). Neuronal plasminogen activators: cell surface binding sites and involvement in neurite outgrowth. J. Neurosci. 9, 4269–4286. 2512375 10.1523/JNEUROSCI.09-12-04269.1989 PMC6569650

[B84] Qian Z. Gilbert M. E. Colicos M. A. Kandel E. R. Kuhl D. (1993). Tissue-plasminogen activator is induced as an immediate-early gene during seizure, kindling and long-term potentiation. Nature 361, 453–457. 10.1038/361453a0 8429885

[B85] Reinhard E. Suidan H. S. Pavlik A. Monard D. (1994). Glia-derived nexin/protease nexin-1 is expressed by a subset of neurons in the rat brain. J. Neurosci. Res. 37, 256–270. 10.1002/jnr.490370211 8151733

[B86] Ricagno S. Caccia S. Sorrentino G. Antonini G. Bolognesi M. (2009). Human neuroserpin: structure and time-dependent inhibition. J. Mol. Biol. 388, 109–121. 10.1016/j.jmb.2009.02.056 19265707

[B87] Rodríguez-González R. Agulla J. Pérez-Mato M. Sobrino T. Castillo J. (2011). Neuroprotective effect of neuroserpin in rat primary cortical cultures after oxygen and glucose deprivation and tPA. Neurochem. Int. 58, 337–343. 10.1016/j.neuint.2010.12.006 21163314

[B88] Rogove A. D. Siao C. Keyt B. Strickland S. Tsirka S. E. (1999). Activation of microglia reveals a non-proteolytic cytokine function for tissue plasminogen activator in the central nervous system. J. Cell Sci. 112 (Pt 22), 4007–4016. 10547361 10.1242/jcs.112.22.4007

[B89] Rogove A. D. Tsirka S. E. (1998). Neurotoxic responses by microglia elicited by excitotoxic injury in the mouse hippocampus. Curr. Biol. 8, 19–25. 10.1016/S0960-9822(98)70016-8 9427623

[B90] Samson A. L. Nevin S. T. Croucher D. Niego B. Daniel P. B. Weiss T. W.. (2008). Tissue-type plasminogen activator requires a co-receptor to enhance NMDA receptor function. J. Neurochem. 107, 1091–1101. 10.1111/j.1471-4159.2008.05687.x 18796005 PMC3198853

[B91] Sappino A. P. Madani R. Huarte J. Belin D. Kiss J. Z. Wohlwend A.. (1993). Extracellular proteolysis in the adult murine brain. J. Clin. Invest. 92, 679–685. 10.1172/JCI116637 8349806 PMC294901

[B92] Sashindranath M. Sales E. Daglas M. Freeman R. Samson A. L. Cops E. J.. (2012). The tissue-type plasminogen activator-plasminogen activator inhibitor 1 complex promotes neurovascular injury in brain trauma: evidence from mice and humans. Brain 135, 3251–3264. 10.1093/brain/aws178 22822039 PMC3501968

[B93] Sawdey M. S. Loskutoff D. J. (1991). Regulation of murine type 1 plasminogen activator inhibitor gene expression *in vivo*. Tissue specificity and induction by lipopolysaccharide, tumor necrosis factor-alpha, and transforming growth factor-beta. J. Clin. Invest. 88, 1346–1353. 10.1172/JCI115440 1918385 PMC295605

[B94] Scott R. W. Bergman B. L. Bajpai A. Hersh R. T. Rodriguez H. Jones B. N.. (1985). Protease nexin. Properties and a modified purification procedure. J. Biol. Chem. 260, 7029–7034. 3997857

[B95] Seeds N. W. Basham M. E. Ferguson J. E. (2003). Absence of tissue plasminogen activator gene or activity impairs mouse cerebellar motor learning. J. Neurosci. 23, 7368–7375. 12917371 10.1523/JNEUROSCI.23-19-07368.2003 PMC6740439

[B96] Seeds N. W. Basham M. E. Haffke S. P. (1999). Neuronal migration is retarded in mice lacking the tissue plasminogen activator gene. Proc. Natl. Acad. Sci. U.S.A. 96, 14118–14123. 10.1073/pnas.96.24.14118 10570208 PMC24200

[B97] Shi Y. Mantuano E. Inoue G. Campana W. M. Gonias S. L. (2009). Ligand binding to LRP1 transactivates Trk receptors by a Src family kinase-dependent pathway. Sci. Signal 2, ra18. 10.1126/scisignal.2000188 19401592 PMC2696635

[B98] Siao C. J. Tsirka S. E. (2002). Tissue plasminogen activator mediates microglial activation via its finger domain through annexin II. J. Neurosci. 22, 3352–3358. 11978811 10.1523/JNEUROSCI.22-09-03352.2002 PMC6758380

[B99] Siconolfi L. B. Seeds N. W. (2001). Mice lacking tPA, uPA, or plasminogen genes showed delayed functional recovery after sciatic nerve crush. J. Neurosci. 21, 4348–4355. 11404420 10.1523/JNEUROSCI.21-12-04348.2001 PMC6762762

[B100] Siconolfi L. B. Seeds N. W. (2003). Mice lacking tissue plasminogen activator and urokinase plasminogen activator genes show attenuated matrix metalloproteases activity after sciatic nerve crush. J. Neurosci. Res. 74, 430–434. 10.1002/jnr.10786 14598319

[B101] Silverman M. A. Johnson S. Gurkins D. Farmer M. Lochner J. E. Rosa P.. (2005). Mechanisms of transport and exocytosis of dense-core granules containing tissue plasminogen activator in developing hippocampal neurons. J. Neurosci. 25, 3095–3106. 10.1523/JNEUROSCI.4694-04.2005 15788766 PMC6725077

[B102] Simonin Y. Charron Y. Sonderegger P. Vassalli J. D. Kato A. C. (2006). An inhibitor of serine proteases, neuroserpin, acts as a neuroprotective agent in a mouse model of neurodegenerative disease. J. Neurosci. 26, 10614–10619. 10.1523/JNEUROSCI.3582-06.2006 17035547 PMC6674675

[B103] Strickland S. (2001). Tissue plasminogen activator in nervous system function and dysfunction. Thromb. Haemost. 86, 138–143. 11486999

[B104] Su E. J. Fredriksson L. Geyer M. Folestad E. Cale J. Andrae J.. (2008). Activation of PDGF-CC by tissue plasminogen activator impairs blood-brain barrier integrity during ischemic stroke. Nat. Med. 14, 731–737. 10.1038/nm1787 18568034 PMC2811427

[B105] Sumii T. Lo E. H. (2002). Involvement of matrix metalloproteinase in thrombolysis-associated hemorrhagic transformation after embolic focal ischemia in rats. Stroke 33, 831–836. 10.1161/hs0302.104542 11872911

[B106] Takao M. Benson M. D. Murrell J. R. Yazaki M. Piccardo P. Unverzagt F. W.. (2000). Neuroserpin mutation S52R causes neuroserpin accumulation in neurons and is associated with progressive myoclonus epilepsy. J. Neuropathol. Exp. Neurol. 59, 1070–1086. 11138927 10.1093/jnen/59.12.1070

[B107] Teesalu T. Kulla A. Simisker A. Sirén V. Lawrence D. A. Asser T.. (2004). Tissue plasminogen activator and neuroserpin are widely expressed in the human central nervous system. Thromb. Haemost. 92, 358–368. 10.1160/th02-12-0310 15269833

[B108] Tsang V. W. K. Young D. During M. J. Birch N. P. (2014). AAV-mediated overexpression of neuroserpin in the hippocampus decreases PSD-95 expression but does not affect hippocampal-dependent learning and memory. PLoS ONE 9:e91050. 10.1371/journal.pone.0091050 24608243 PMC3946662

[B109] Tsirka S. E. Gualandris A. Amaral D. G. Strickland S. (1995). Excitotoxin-induced neuronal degeneration and seizure are mediated by tissue plasminogen activator. Nature 377, 340–344. 10.1038/377340a0 7566088

[B110] Tsirka S. E. Rogove A. D. Bugge T. H. Degen J. L. Strickland S. (1997). An extracellular proteolytic cascade promotes neuronal degeneration in the mouse hippocampus. J. Neurosci. 17, 543–552. 8987777 10.1523/JNEUROSCI.17-02-00543.1997 PMC6573220

[B111] Valiente M. Obenauf A. C. Jin X. Chen Q. Zhang X. H. Lee D. J.. (2014). Serpins promote cancer cell survival and vascular co-option in brain metastasis. Cell 156, 1002–1016. 10.1016/j.cell.2014.01.040 24581498 PMC3988473

[B112] Vanlandingham J. W. Cekic M. Cutler S. M. Hoffman S. W. Washington E. R. Johnson S. J.. (2008). Progesterone and its metabolite allopregnanolone differentially regulate hemostatic proteins after traumatic brain injury. J. Cereb. Blood Flow Metab. 28, 1786–1794. 10.1038/jcbfm.2008.73 18628783

[B113] Vawter M. P. Shannon Weickert C. Ferran E. Matsumoto M. Overman K. Hyde T. M.. (2004). Gene expression of metabolic enzymes and a protease inhibitor in the prefrontal cortex are decreased in schizophrenia. Neurochem. Res. 29, 1245–1255. 10.1023/B:NERE.0000023611.99452.47 15176481

[B114] Wang X. Lee S. R. Arai K. Lee S. R. Tsuji K. Rebeck G. W.. (2003). Lipoprotein receptor-mediated induction of matrix metalloproteinase by tissue plasminogen activator. Nat. Med. 9, 1313–1317. 10.1038/nm926 12960961

[B115] Wang X. Tsuji K. Lee S. R. Ning M. Furie K. L. Buchan A. M.. (2004). Mechanisms of hemorrhagic transformation after tissue plasminogen activator reperfusion therapy for ischemic stroke. Stroke 35, 2726–2730. 10.1161/01.STR.0000143219.16695.af 15459442

[B116] Wang Y. F. Tsirka S. E. Strickland S. Stieg P. E. Soriano S. G. Lipton S. A. (1998). Tissue plasminogen activator (tPA) increases neuronal damage after focal cerebral ischemia in wild-type and tPA-deficient mice. Nat. Med. 4, 228–231. 10.1038/nm0298-228 9461198

[B117] Wannier-Morino P. Rager G. Sonderegger P. Grabs D. (2003). Expression of neuroserpin in the visual cortex of the mouse during the developmental critical period. Eur. J. Neurosci. 17, 1853–1860. 10.1046/j.1460-9568.2003.02628.x 12752785

[B118] Ware J. H. Dibenedetto A. J. Pittman R. N. (1995). Localization of tissue plasminogen activator mRNA in adult rat brain. Brain Res. Bull. 37, 275–281. 10.1016/0361-9230(95)00008-3 7627570

[B119] Woo N. H. Teng H. K. Siao C. J. Chiaruttini C. Pang P. T. Milner T. A.. (2005). Activation of p75NTR by proBDNF facilitates hippocampal long-term depression. Nat. Neurosci. 8, 1069–1077. 10.1038/nn1510 16025106

[B120] Wu F. Echeverry R. Wu J. An J. Haile W. B. Cooper D. S.. (2013). Tissue-type plasminogen activator protects neurons from excitotoxin-induced cell death via activation of the ERK1/2-CREB-ATF3 signaling pathway. Mol. Cell. Neurosci. 52, 9–19. 10.1016/j.mcn.2012.10.001 23063501 PMC3540185

[B121] Wu F. Wu J. Nicholson A. D. Echeverry R. Haile W. B. Catano M.. (2012). Tissue-type plasminogen activator regulates the neuronal uptake of glucose in the ischemic brain. J. Neurosci. 32, 9848–9858. 10.1523/JNEUROSCI.1241-12.2012 22815500 PMC3437989

[B122] Wu J. Echeverry R. Guzman J. Yepes M. (2010). Neuroserpin protects neurons from ischemia-induced plasmin-mediated cell death independently of tissue-type plasminogen activator inhibition. Am. J. Pathol. 177, 2576–2584. 10.2353/ajpath.2010.100466 20864675 PMC2966813

[B123] Wu Y. P. Siao C. J. Lu W. Sung T. C. Frohman M. A. Milev P.. (2000). The tissue plasminogen activator (tPA)/plasmin extracellular proteolytic system regulates seizure-induced hippocampal mossy fiber outgrowth through a proteoglycan substrate. J. Cell Biol. 148, 1295–1304. 10.1083/jcb.148.6.1295 10725341 PMC2174310

[B124] Yamada M. Takahashi K. Ukai W. Hashimoto E. Saito T. Yamada M. (2010). Neuroserpin is expressed in early stage of neurogenesis in adult rat hippocampus. Neuroreport 21, 138–142. 10.1097/WNR.0b013e3283350b24 20010310

[B125] Yepes M. Sandkvist M. Moore E. G. Bugge T. H. Strickland D. K. Lawrence D. A. (2003). Tissue-type plasminogen activator induces opening of the blood-brain barrier via the LDL receptor-related protein. J. Clin. Invest. 112, 1533–1540. 10.1172/JCI200319212 14617754 PMC259131

[B126] Yepes M. Sandkvist M. Wong M. K. Coleman T. A. Smith E. Cohan S. L.. (2000). Neuroserpin reduces cerebral infarct volume and protects neurons from ischemia-induced apoptosis. Blood 96, 569–576. 10887120

[B127] Yi J. S. Kim Y. H. Koh J. Y. (2004). Infarct reduction in rats following intraventricular administration of either tissue plasminogen activator (tPA) or its non-protease mutant S478A-tPA. Exp. Neurol. 189, 354–360. 10.1016/j.expneurol.2004.05.032 15380485

[B128] Zhang C. An J. Strickland D. K. Yepes M. (2009). The low-density lipoprotein receptor-related protein 1 mediates tissue-type plasminogen activator-induced microglial activation in the ischemic brain. Am. J. Pathol. 174, 586–594. 10.2353/ajpath.2009.080661 19147818 PMC2630566

[B129] Zhang Z. Zhang L. Yepes M. Jiang Q. Li Q. Arniego P.. (2002). Adjuvant treatment with neuroserpin increases the therapeutic window for tissue-type plasminogen activator administration in a rat model of embolic stroke. Circulation 106, 740–745. 10.1161/01.CIR.0000023942.10849.41 12163437

[B130] Zhuo M. Holtzman D. M. Li Y. Osaka H. DeMaro J. Jacquin M.. (2000). Role of tissue plasminogen activator receptor LRP in hippocampal long-term potentiation. J. Neurosci. 20, 542–549. 10632583 10.1523/JNEUROSCI.20-02-00542.2000 PMC6772406

[B131] Zou T. Ling C. Xiao Y. Tao X. Ma D. Chen Z. L.. (2006). Exogenous tissue plasminogen activator enhances peripheral nerve regeneration and functional recovery after injury in mice. J. Neuropathol. Exp. Neurol. 65, 78–86. 10.1097/01.jnen.0000195942.25163.f5 16410751

